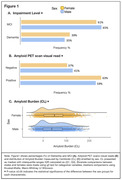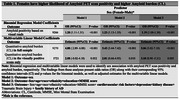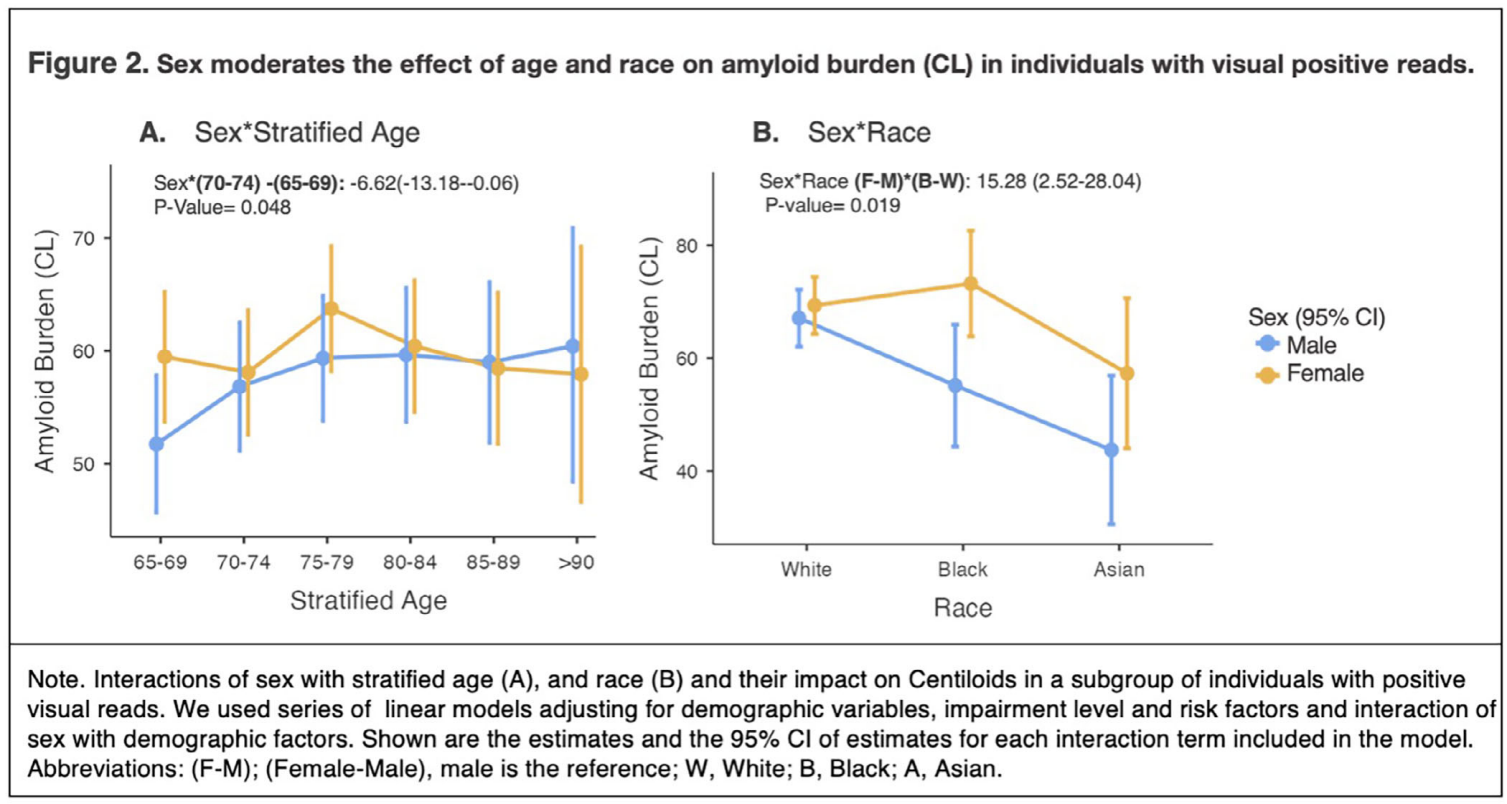# Sex Differences in Amyloid PET in a Large, Real‐Life Sample from the Imaging Dementia‐Evidence for Amyloid Scanning (IDEAS) Study

**DOI:** 10.1002/alz.090224

**Published:** 2025-01-09

**Authors:** Maison Abu Raya, Ehud Zeltzer, Daniel R. Schonhaut, Isabel Elaine Allen, Maria C. Carrillo, Constantine Gatsonis, Lucy Hanna, Bruce E Hillner, Leo Iaccarino, Andrew March, Nidhi S Mundada, Jhony Alejandro Mejía‐Perez, Barry A. Siegel, Charles Windon, Rachel A. Whitmer, Renaud La Joie, Gil D. Rabinovici

**Affiliations:** ^1^ University of california San Francisco, San Francisco, CA USA; ^2^ Memory and Aging Center, Weill Institute for Neurosciences, University of California, San Francisco, San Francisco, CA USA; ^3^ Department of Epidemiology and Biostatistics, University of California, San Francisco, San Francisco, CA USA; ^4^ Global Brain Health Institute, University of California San Francisco, San Francisco, CA USA; ^5^ Memory and Aging Center, UCSF Weill Institute for Neurosciences, University of California, San Francisco, San Francisco, CA USA; ^6^ University of California San Francisco, San Francisco, CA USA; ^7^ Alzheimer's Association, Chicago, IL USA; ^8^ Brown University, Providence, RI USA; ^9^ Virginia Commonwealth University, Richmond, VA USA; ^10^ University of California, San Francisco, San Francisco, CA USA; ^11^ American College of Radiology, Reston, VA USA; ^12^ Latin American Brain Health Institute (BrainLat), Universidad Adolfo Ibañez, Santiago de Chile Chile; ^13^ Mallinckrodt Institute of Radiology, Washington University School of Medicine, St. Louis, MO USA; ^14^ University of California, Davis School of Medicine, Sacramento, CA USA; ^15^ Weill Institute for Neurosciences, University of California, San Francisco, San Francisco, CA USA

## Abstract

**Background:**

Previous studies on sex differences in amyloid burden have shown inconsistent findings. We examined the effect of sex on amyloid‐PET outcomes in a large, real‐world, cohort of individuals with cognitive impairment.

**Method:**

The IDEAS study evaluated the clinical utility of amyloid‐PET in 18,295 Medicare beneficiaries age ≥65 years with MCI or dementia. All scans were visually interpreted as positive or negative at each site by a local radiologist or nuclear medicine physician. A subset of 10,361 scans were centrally processed and quantified in Centiloids. We used multivariate logistic regression to calculate odds ratios of amyloid‐PET positivity (based on visual read) for males and females, adjusting for demographic and clinical risk factors. We used linear regression to assess the association between sex and amyloid burden quantified in Centiloids.

**Result:**

Of 10,361 included individuals, 51% were females. Compared to males, females were slightly younger (75 versus 76 median age, p=0.008) and had higher rates of dementia (39.3% versus 35.2%, p<0.001). Rates of vascular risk factors were significantly higher in males than females, whereas females had significantly higher rates of history of depression and family history of AD. Females had higher rates of amyloid‐PET positivity than males (63% versus 59%, p<.001) and higher Centiloid values (median=48.7 versus 36.9, p<.01); see Figure 1 and Table 1 model 1. Sex differences remained significant in models adjusted for demographics and clinical risk factors (Table 1, models 2‐3). In an analysis that included only individuals with visually positive amyloid‐PET, females exhibited higher Centiloid values than males (Table 1 multivariable linear models). In amyloid‐positive individuals, we found a significant interaction between sex and age, with greatest sex differences in amyloid burden found in the youngest females (Figure 2A). We also found a significant interaction between sex and race, with greatest differences found in Black females vs. males (Figure 2B).

**Conclusion:**

Females with cognitive impairment exhibited a higher frequency of amyloid‐PET positivity and higher amyloid burden. Our findings shed light on sex‐specific biological and potential sociocultural differences in Alzheimer's disease pathology.